# 3D-QSAR Investigation of Synthetic Antioxidant Chromone Derivatives by Molecular Field Analysis

**DOI:** 10.3390/ijms9030235

**Published:** 2008-02-29

**Authors:** Weerasak Samee, Patcharawee Nunthanavanit, Jiraporn Ungwitayatorn

**Affiliations:** 1 Department of Pharmaceutical Chemistry and Pharmacognosy, Faculty of Pharmacy, Srinakharinwirot University, Nakhon Nayok, 26120, Thailand; E-mail: patcharawee@swu.ac.th; 2 Department of Pharmaceutical Chemistry, Faculty of Pharmacy, Mahidol University, Bangkok, 10400, Thailand; E-mail: pyjuw@mahidol.ac.th

**Keywords:** 3D-QSAR, Chromone, Molecular field analysis (MFA), Antioxidants, Genetic partial least squares (G/PLS) method

## Abstract

A series of 7-hydroxy, 8-hydroxy and 7,8-dihydroxy synthetic chromone derivatives was evaluated for their DPPH free radical scavenging activities. A training set of 30 synthetic chromone derivatives was subject to three-dimensional quantitative structure-activity relationship (3D-QSAR) studies using molecular field analysis (MFA). The substitutional requirements for favorable antioxidant activity were investigated and a predictive model that could be used for the design of novel antioxidants was derived. Regression analysis was carried out using genetic partial least squares (G/PLS) method. A highly predictive and statistically significant model was generated. The predictive ability of the developed model was assessed using a test set of 5 compounds (*r*^2^_pred_ = 0.924). The analyzed MFA model demonstrated a good fit, having *r*^2^ value of 0.868 and cross-validated coefficient *r*^2^_cv_ value of 0.771.

## 1. Introduction

**Figure 1.**
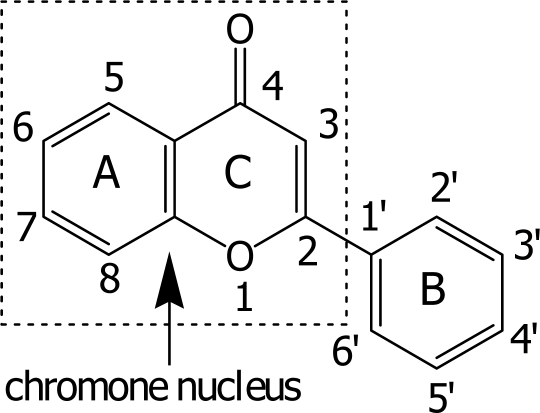
General flavone structure.

Free radicals containing major species of reactive oxygen species (ROS) and/or reactive nitrogen species (RNS) are generated inside the living cells by exposure to several endogenous and exogenous agents. They are known to cause permanent damages to biomolecules as implicated in several diseases or symptoms e.g. arteriosclerosis, Alzheimer’s disease, cancer, and even aging [1–6]. Consequently, free radical scavengers are considered to be prospects as protective or therapeutic agents against such diseases. Flavonoids are naturally occurring compounds in a class of benzo-γ-pyrone derivatives ubiquitously found in plants. They possess a wide spectrum of biological activities. Some flavonoids have been reported to possess anticancer, anti HIV, anti-inflammatory, and several other activities [7–9]. Recent interests in these substances have been stimulated by potential health benefits arising from the antioxidant activity of polyphenolic flavonoids [10, 11]. These are the result of their high propensity to transfer electrons, to chelate ferrous ions, and to scavenge reactive oxygen species [12–13]. Structure-activity relationship (SAR) studies of natural flavonoids demonstrate that the dissociation of hydroxyl functions occurs in the following sequence: 7-OH > 4′-OH > 5-OH [14]. The *o*-dihydroxy structure in the B ring, the 2,3-double bond in conjugation with the 4-oxo function in the C ring of flavone (Figure 1), and the 3- and 5-OH groups with the 4-oxo function in A and C rings are essential for effective free radical scavenging activity [15].

The chromone derivatives were synthesized and tested for their radical scavenging activities. Substitution of benzoyl group at position 3 in some compounds increased the number of conjugation bonds and improved the radical stabilization of flavonoids [16]. A number of SAR and QSAR studies have been performed on the antioxidant activity of natural chromone derivatives [17–22]. However, synthetic chromone derivatives have not been investigated. Therefore, the aim of this study was to explore the substitutional requirements of synthetic chromone derivatives as antioxidants to obtain a highly predictive 3D-QSAR model. 3D-QSAR analysis was performed using the most widely used computational tool, molecular field analysis (MFA) with respect to the steric and electrostatic influences.

MFA is a method implemented in the Cerius^2^ program. Its formalism calculates probe interaction energies on a rectangular grid around a bundle of active molecules. The surface is generated from a “shape field.” The atomic coordinates of the contributing models are used to compute field values on each point of a 3D grid. MFA then evaluates the energy between a probe (H^+^, CH_3_, and donor/acceptor) and a molecular model at a series of points defined by a rectangular grid. Fields of molecules are represented with grids in MFA and corresponding energy associated with an MFA grid point can serve as input for the calculation of a QSAR. These energies are added to the study table to form new columns headed according to the probe type. Because of the large number of points used as independent variables, genetic partial least squares (G/PLS) is generally used to derive the QSAR models [23]. The best model is selected based on statistical measures such as data points (n), correlation coefficient (*r*), square correlation coefficient (*r*^2^), cross-validated correlation coefficient (*r*^2^_cv_), predicted correlation coefficient (*r*^2^_pred_), predicted sum of squares (PRESS), bootstrap correlation coefficient (*r*^2^_BS_) and least-square error of fit (LSE).

In this study, we evaluated the *in vitro* free radical scavenging activities of chromone derivatives by DPPH assay. Molecular field analysis (MFA) was carried out on a set of 36 synthetic chromone derivatives.

## 2. Results and Discussion

### 2.1 Structure-radical scavenging activity relationship

**Figure 2.**
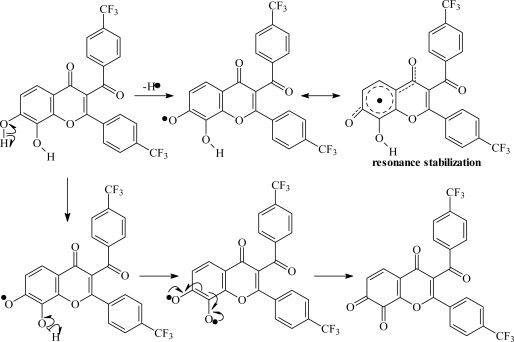
Resonance stabilization of a hydroxyl chromone molecule proposed for radical scavenging activity.

Thirty-six synthetic chromone derivatives (indicated as compounds **1–36**) were assessed for their antioxidant activities by DPPH radical scavenging assay. As shown in Tables 1 and 2, various chromones exhibited different levels of activity, ranging from EC_50_ = 2.58 to 182.77 μM which are more potent than the well known natural antioxidants, e.g., quercetin and luteolin which possessed IC_50_ = 10.89 and 11.04 μM, respectively [24]. Structure-radical scavenging activity relationship demonstrated that the 7,8-dihydroxy-2-phenyl-3-benzoyl substituted compounds (compounds **29**, **30** and **36**) exhibited a strong antioxidant activity with low log EC_50_. This indicated that dihydroxy substitution (cathecol group) on ring A was essential for radical scavenging activity. The presence of benzoyl group at position 3 confers a high degree of stability toward the phenoxy radicals by participating in electron delocalization and thus is an important feature for potential antiradical property. The proposed model for the progression of successive dehydrogenation from a hydroxyl chromone molecule using adjacent OH-containing aromatic ring is shown in Figure 2. The initial dehydrogenation occurs on the *para*-OH group. If this is the case, prototropy from an adjacent OH group will be easy. This semiquinone type radical is more reactive than the original phenol molecule, so the second hydrogen liberation proceeds rapidly, thus resulting in biradical changes into a quinone.

### 2.2 3D-QSAR modeling

**Figure 3.**
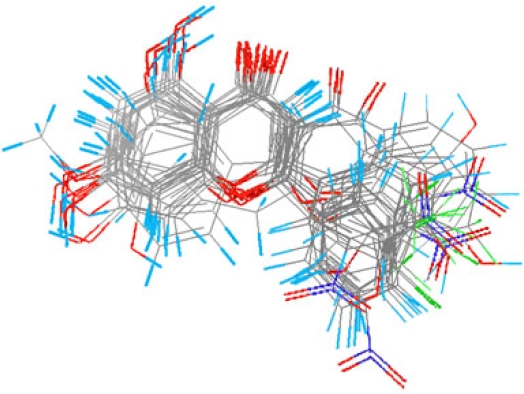
Superimposition of the aligned molecules in the training set.

The MFA model of 35 chromone derivatives (30 compounds in a training set; 5 compounds in a test set) was developed using field fit alignment. The most active compound, 7,8-dihydroxy-2-(4′-trifluoromethylphenyl)-3-(4″-trifluoromethylbenzoyl)chromone **29** was used as a template model for superimposing the rest of the molecules. Superimposition of the aligned molecules is shown in Figure 3.

**Figure 4.**
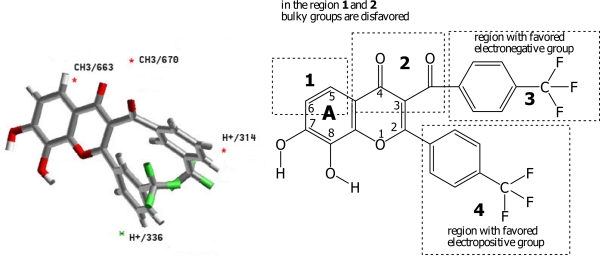
Mapping of the best MFA model and the interaction points. The most active compound, 7,8-dihydroxy-2-(4′-trifluoromethylphenyl)-3-(4″-trifluoromethylbenzoyl)chromone **29**, is displayed in background as reference.

The steric (CH_3_) and electrostatic (H^+^) descriptors in the MFA-QSAR equations specify the regions where variations in the structural features (steric or electrostatic) of different compounds in the training set, leading to either an increase or a decrease in activities. The steric descriptor with positive or negative coefficients shows a region where bulky substituent is favored or disfavored, respectively. The electrostatic descriptor with a positive coefficient indicates a region favorable for electropositive group, while a negative coefficient indicates that an electronegative (electron-withdrawing) group is required at the position. The numbers accompanying descriptors in the equations represent their positions in the three-dimensional MFA grid (Figure 4). The MFA-QSAR equation is expressed as follow:

(1) $$\begin{array}{ll} \text{Activity}=2.86587 & -0.021102\;({\text{H}}^{+}/314)\\ & -0.004635\;(\text{Vm})\\ & +0.010606\;({\text{H}}^{+}/336)\\ & -0.01133({\text{CH}}_{3}/663)\\ & -0.009739({\text{CH}}_{3}/670) \end{array}$$

n = 30, *r* = 0.932, *r*^2^ = 0.868, *r*^2^_cv_ = 0.771, PRESS = 1.022, *r*^2^_BS_ = 0.857, LSE = 0.02, N = 5, *r*^2^_pred_ = 0.924

The presence of two steric descriptors (CH_3_/663) and (CH_3_/670) with negative coefficients, indicates that bulky substituents are disfavored. The presence of electrostatic descriptor (H^+^/336) with a positive coefficient on phenyl ring suggests that electropositive groups are favored, while (H^+^/314) with negative coefficients indicates that electronegative groups should be substituted on benzoyl ring. Appearance of descriptor (Vm) with a negative coefficient demonstrates that larger molecule decrease activity. Figure 4 illustrates the regions around the molecule (compound **29**) corresponding to the MFA model. This proposed model can be accounted for the lowest activity of compound **31** (EC_50_ = 182.77) which possessed no electronegative group in region 3 and electropositive group in region 4.

A QSAR equation is generally acceptable if the correlation coefficient (*r*) is approximately 0.9 or higher. The *r* value is a relative measure of the quality of fit of the model. Its value depends on the overall variance of the data. An *r*^2^_cv_, a squared correlation coefficient generated during a cross-validation procedure, is used as a diagnostic tool to evaluate the predictive power of an equation. Cross-validation is often used to determine how large a model (number of terms) can be used for a given data set. Equation 1 explains 86.8% variance in the activity with respect to the steric and electrostatic fields and molecular volume while leave-one-out cross-validation power of prediction was found to be 77.1%. An *r*^2^_BS_ value of 0.857 is an average squared correlation coefficient calculated during the validation procedure. The predictive power of the model was calculated by using the following equation

(2) $${r^{2}}_{\text{pred}}=\frac{\left(\text{SD}-\text{PRESS}\right)}{\text{SD}}$$

where SD is the sum of the squared deviations between the biological activities of each molecules and the mean activity of the training set of molecules and PRESS is the sum of squared deviations between the predicted and actual activity values for every molecule in the test set.

**Figure 5.**
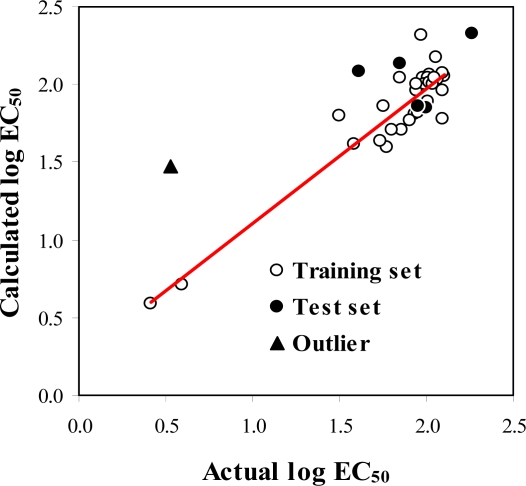
Plot of actual *versus* calculated log EC_50_ of the training and test set molecules.

**Figure 6.**
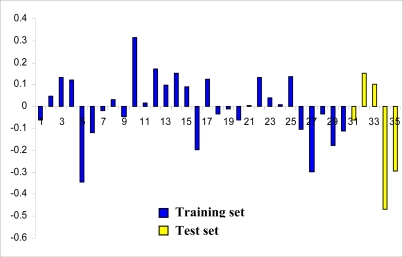
Plot of residuals for training set and test set molecules.

The calculated activity obtained from equation 1 and actual activity of the training set and test set molecules are summarized in Table 1 and 2. Scattered plots of calculated and actual activities and the plot of residuals for the training set and the test set molecules are shown in Figure 5 and 6, respectively. Most of the molecules showed residual values less than 0.2. The outlier molecule was 7,8-dihydroxy-2-(4′-nitrophenyl)-3-(4″-nitrobenzoyl)chromone **36** (residual = 0.947). The obtained MFA model shows good statistical results with *r*^2^_cv_ = 0.771, conventional *r*^2^ = 0.868 and *r*^2^_pred_ = 0.924.

## 3. Conclusions

MFA-QSAR studies were performed on a series of synthetic chromone derivatives using field fit alignment with high predictive ability, high cross-validated, conventional and predictive *r*^2^. The MFA equation suggested that electronegative group on benzoyl ring and the electropositive group on phenyl ring play an important role for antioxidant activity. These electronegative and electropositive substituents might help in the radical stabilization throughout the chromone nucleus. The steric descriptors indicated that the bulky substituents near position 5 and chromone carbonyl were disfavored. Steric hindrance around these regions may interfere with the planarity between ring A and carbonyl group of the chromone nucleus, therefore affecting radical delocalization shown in Figure 2.

## 4. Experimental Section

### 4.1 Structures and Biological data

Chromone derivatives were synthesized by one-pot cyclization reaction with 1,8-diazabicyclo[5,4,0]undec-7-ene (DBU) as catalyst [25]. The antioxidant activities of the synthesized compounds were assessed on the basis of the radical scavenging effect on the DPPH free radicals as described previously [16]. The concentrations of test samples required to scavenge 50% of DPPH free radicals (EC_50_ μM) were converted into corresponding log EC_50_ values.

### 4.2 Molecular structure generation

The molecular structures of chromone derivatives were modeled with SYBYL 7.0 molecular modeling program (Tripos Associates, Saint Louis, MO) on an Indigo Elan workstation (Silicon Graphics Inc., Mountain View, CA) using the sketch approach. The fragment libraries in SYBYL database were used as building blocks for construction of larger images. Firstly, each structure was energy minimized using the standard Tripos force field (Powell method and 0.05 kcal/mol.Å energy gradient convergence criteria) and electrostatic charge was assigned by the Gasteiger-Hückel method. Further, geometry optimization was then carried out with the MOPAC 6 package using the semi-empirical PM3 with Gasteiger-Hückel for charges calculation. The SMILESes forms of all structures are shown in Table 3.

### 4.3 Structural alignments

The field fit alignment method was used for MFA. All molecules were submitted to the CONFORMER SEARCH module within Cerius^2^ to generate 150 conformers of each molecule using Boltzman jump method [26]. The lowest energy conformer of each molecule was selected. All the selected conformers were aligned using field fit alignment method in the QSAR module. The most active compound, 7,8-dihydroxy-2-(4′-trifluoromethylphenyl)-3-(4″-trifluoromethylbenzoyl)chromone **29**, was used as a template model for superimposing the rest of the molecules.

### 4.4 Molecular field analysis (MFA)

MFA studies were performed with the QSAR module of Cerius^2^. The molecular field was created using CH_3_ and H^+^ as probes representing steric and electrostatic fields, respectively. The steric and electrostatic fields were sampled at each point of regularly spaced grid of 2 Å. In addition, numerous spatial and structural descriptors such as polarizability, dipole moment, radius of gyration, molecular area, molecular dimension, density, principal moment of inertia, molecular volume, molecular weight, number of rotatable bonds, hydrogen bond donors and acceptors, log P, molar refractivity and others were also calculated and considered as independent variables. Only 10% of the total descriptors with the highest variance were considered for further analysis. Regression analysis was carried out using genetic partial least squares (G/PLS) method consisting of 5000 generations with a population size of 100. The optimum number of components was set to 4 based on better *r*^2^ and *r*^2^_cv_ values for a given training set. An energy cutoff of ± 30.0 kcal/mol was set for both steric and electrostatic contributions. The smoothing parameter, d, was set to 1.0 to control the bias in the scoring factors between equations with different number of terms. The length of the final equation was fixed to five descriptors. The linear option was used in the equation creation. Cross validation was performed with the leave-one-out procedure. The PLS analysis was set to no scale.

## Figures and Tables

**Table 1.** Molecular structures and corresponding antioxidant activities of synthetic chromones (Training set).

**Figure:**
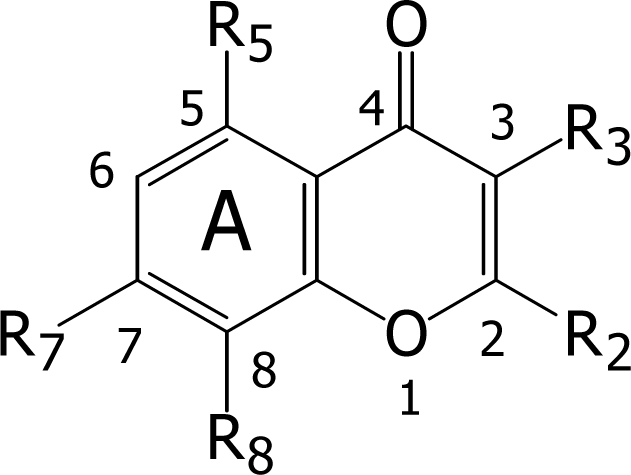

Cpd	R_2_	R_3_	R_5_	R_7_	R_8_	EC_50_ (μM)	log EC_50_		Residuals
							Actual	Calculated	
**1**	Phenyl	H	H	H	OH	96.18	1.983	2.046	0.063
**2**	Phenyl	H	H	OH	H	125.62	2.099	2.053	0.046
**3**	Benzyl	H	H	OH	H	125.09	2.097	1.964	0.133
**4**	4′-(NO_2_)Phenyl	H	H	OH	H	101.82	2.008	1.888	0.120
**5**	3′-(CF_3_)-Phenyl	H	H	OH	H	93.37	1.970	2.315	−0.345
**6**	4′-(F)-Phenyl	H	H	OH	H	113.22	2.054	2.172	−0.118
**7**	3′,5′-(diNO_2_)-Phenyl	H	H	OH	H	87.45	1.942	1.962	−0.020
**8**	3′-(Cl)-Phenyl	H	H	OH	H	117.08	2.068	2.035	0.034
**9**	4′-(*t*-butyl)-Phenyl	H	H	OH	H	104.56	2.019	2.064	−0.045
**10**	Phenyl	CH_3_	H	OH	H	124.19	2.094	1.780	0.314
**11**	Benzyl	CH_3_	H	OH	H	123.47	2.092	2.074	0.018
**12**	4′-(NO_2_)-Phenyl	4″-(NO_2_)-Benzoyl	H	OH	H	59.30	1.773	1.600	0.173
**13**	3′-(CF_3_)-Phenyl	3″-(CF_3_)-Benzoyl	H	OH	H	54.32	1.735	1.637	0.098
**14**	4′-(F)-Phenyl	4″-(F)-Benzoyl	H	OH	H	72.53	1.860	1.709	0.151
**15**	3′,4′-(diF)-Phenyl	3″,4″-(diF)-Benzoyl	H	OH	H	63.00	1.799	1.710	0.089
**16**	4′-(OCH_3_)-Phenyl	4″-(OCH_3_)-Benzoyl	H	OH	H	70.80	1.850	2.047	−0.197
**17**	3′-(CF_3_)-Phenyl	H	OH	OH	H	85.46	1.932	1.806	0.126
**18**	4′-(F)-Phenyl	H	OH	OH	H	102.21	2.010	2.043	−0.034
**19**	3′,4′-(diF)-Phenyl	H	OH	OH	H	98.53	1.994	2.005	−0.016
**20**	4′-(*t*-butyl)-Phenyl	H	OH	OH	H	87.36	1.941	2.002	−0.061
**21**	3′-(Cl)Phenyl	H	OH	OH	H	104.30	2.018	2.014	0.004
**22**	3′,4′-(diCl)-Phenyl	H	OH	OH	H	90.19	1.955	1.823	0.132
**23**	4′-(OCH_3_)-Phenyl	H	OH	OH	H	109.37	2.039	1.999	0.040
**24**	3′-(OCH_3_)-Phenyl	H	OH	OH	H	111.62	2.048	2.040	0.008
**25**	3′,5′-(diNO_2_)-Phenyl	H	OH	OH	H	79.74	1.902	1.766	0.136
**26**	4′-(NO_2_)-Phenyl	4″-(NO_2_)-Benzoyl	OH	OH	H	57.54	1.756	1.860	−0.104
**27**	Phenyl	H	H	OH	OH	31.89	1.504	1.803	−0.300
**28**	Benzyl	H	H	OH	OH	38.12	1.581	1.615	−0.034
**29**	3′-(CF_3_)-Phenyl	3″-(CF_3_)-Benzoyl	H	OH	OH	2.58	0.417	0.589	−0.177
**30**	4′-(F)-Phenyl	4″-(F)-Benzoyl	H	OH	OH	3.93	0.594	0.707	−0.113

**Table 2.** Structures and their corresponding antioxidant activities of synthetic chromones (Test set);* An outlier compound

Cpd	R_2_	R_3_	R_5_	R_7_	R_8_	EC_50_ (μM)	log EC_50_		Residuals
							Actual	Calculated	
**31**	CH_3_	H	H	OH	H	182.77	2.262	2.324	−0.062
**32**	3′,4′-(diCl)-Phenyl	H	H	OH	H	100.22	2.001	1.850	0.151
**33**	4′-(NO_2_)-Phenyl	H	H	OH	H	90.43	1.956	1.856	0.100
**34**	CH_3_	H	H	OH	OH	41.25	1.616	2.084	−0.468
**35**	3′-(OCH_3_)-Phenyl	3″-(OCH_3_)-Benzoyl	H	OH	H	70.31	1.847	2.139	−0.292
36*	4′-(NO_2_)-Phenyl	4″-(NO_2_)-Benzoyl	H	OH	OH	3.37	0.528	1.475	0.947

**Table 3.** The SMILESes forms of the synthetic chromone structures.

Cpd	SMILESes form of structures
1	[H]/C2=C(/Oc1c(O[H])c([H])c([H])c([H])c1C2=O)c3c([H])c([H])c([H])c([H])c3[H]
2	[H]/C2=C(/Oc1c([H])c(O[H])c([H])c([H])c1C2=O)c3c([H])c([H])c([H])c([H])c3[H]
3	[H]/C2=C(/Oc1c([H])c(O[H])c([H])c([H])c1C2=O)C([H])([H])c3c([H])c([H])c([H])c([H])c3[H]
4	[H]/C2=C(/Oc1c([H])c(O[H])c([H])c([H])c1C2=O)c3c([H])c([H])c(N(=O)=O)c([H])c3[H]
5	[H]/C2=C(/Oc1c([H])c(O[H])c([H])c([H])c1C2=O)c3c([H])c([H])c([H])c(c3[H])C([F])([F])[F]
6	[H]/C2=C(/Oc1c([H])c(O[H])c([H])c([H])c1C2=O)c3c([H])c([H])c([F])c([H])c3[H]
7	[H]/C2=C(/Oc1c([H])c(O[H])c([H])c([H])c1C2=O)c3c([H])c(N(=O)=O)c([H])c(N(=O)=O)c3[H]
8	[H]/C2=C(/Oc1c([H])c(O[H])c([H])c([H])c1C2=O)c3c([H])c([H])c([H])c([Cl])c3[H]
9	[H]/C2=C(/Oc1c([H])c(O[H])c([H])c([H])c1C2=O)c3c([H])c([H])c(c([H])c3[H])C(C([H])([H])[H])(C([H])([H])[H])C([H])([H])[H]
10	O=C1C(=C(Oc2c([H])c(O[H])c([H])c([H])c12)c3c([H])c([H])c([H])c([H])c3[H])C([H])([H])[H]
11	O=C1C(=C(Oc2c([H])c(O[H])c([H])c([H])c12)C([H])([H])c3c([H])c([H])c([H])c([H])c3[H])C([H])([H])[H]
12	O=C2C(C(=O)c1c([H])c([H])c(N(=O)=O)c([H])c1[H])=C(Oc3c([H])c(O[H])c([H])c([H])c23)c4c([H])c([H])c(N(=O)=O)c([H])c4[H]
13	O=C2C(C(=O)c1c([H])c(c([H])c([H])c1[H])C([F])([F])[F])=C(Oc3c([H])c(O[H])c([H])c([H])c23)c4c([H])c([H])c([H])c(c4[H])C([F])([F])[F]
14	O=C2C(C(=O)c1c([H])c([H])c([F])c([H])c1[H])=C(Oc3c([H])c(O[H])c([H])c([H])c23)c4c([H])c([H])c([F])c([H])c4[H]
15	O=C2C(C(=O)c1c([H])c([F])c([F])c([H])c1[H])=C(Oc3c([H])c(O[H])c([H])c([H])c23)c4c([H])c([H])c([F])c([F])c4[H]
16	O=C2C(C(=O)c1c([H])c([H])c(OC([H])([H])[H])c([H])c1[H])=C(Oc3c([H])c(O[H])c([H])c([H])c23)c4c([H])c([H])c(OC([H])([H])[H])c([H])c4[H]
17	[H]/C2=C(/Oc1c([H])c(O[H])c([H])c(O[H])c1C2=O)c3c([H])c([H])c([H])c(c3[H])C([F])([F])[F]
18	[H]/C2=C(/Oc1c([H])c(O[H])c([H])c(O[H])c1C2=O)c3c([H])c([H])c([F])c([H])c3[H]
19	[H]/C2=C(/Oc1c([H])c(O[H])c([H])c(O[H])c1C2=O)c3c([H])c([H])c([F])c([F])c3[H]
20	[H]/C2=C(/Oc1c([H])c(O[H])c([H])c(O[H])c1C2=O)c3c([H])c([H])c(c([H])c3[H])C(C([H])([H])[H])(C([H])([H])[H])C([H])([H])[H]
21	[H]/C2=C(/Oc1c([H])c(O[H])c([H])c(O[H])c1C2=O)c3c([H])c([H])c([H])c([Cl])c3[H]
22	[H]/C2=C(/Oc1c([H])c(O[H])c([H])c(O[H])c1C2=O)c3c([H])c([H])c([Cl])c([Cl])c3[H]
23	[H]/C2=C(/Oc1c([H])c(O[H])c([H])c(O[H])c1C2=O)c3c([H])c([H])c(OC([H])([H])[H])c([H])c3[H]
24	[H]/C2=C(/Oc1c([H])c(O[H])c([H])c(O[H])c1C2=O)c3c([H])c(OC([H])([H])[H])c([H])c([H])c3[H]
25	[H]/C2=C(/Oc1c([H])c(O[H])c([H])c(O[H])c1C2=O)c3c([H])c(N(=O)=O)c([H])c(N(=O)=O)c3[H]
26	O=C2C(C(=O)c1c([H])c([H])c(N(=O)=O)c([H])c1[H])=C(Oc3c([H])c(O[H])c([H])c(O[H])c23)c4c([H])c([H])c(N(=O)=O)c([H])c4[H]
27	[H]/C2=C(/Oc1c(O[H])c(O[H])c([H])c([H])c1C2=O)c3c([H])c([H])c([H])c([H])c3[H]
28	[H]/C2=C(/Oc1c(O[H])c(O[H])c([H])c([H])c1C2=O)C([H])([H])c3c([H])c([H])c([H])c([H])c3[H]
29	O=C2C(C(=O)c1c([H])c(c([H])c([H])c1[H])C([F])([F])[F])=C(Oc3c(O[H])c(O[H])c([H])c([H])c23)c4c([H])c([H])c([H])c(c4[H])C([F])([F])[F]
30	O=C2C(C(=O)c1c([H])c([H])c([F])c([H])c1[H])=C(Oc3c(O[H])c(O[H])c([H])c([H])c23)c4c([H])c([H])c([F])c([H])c4[H]
31	[H]/C2=C(/Oc1c([H])c(O[H])c([H])c([H])c1C2=O)C([H])([H])[H]
32	[H]/C2=C(/Oc1c([H])c(O[H])c([H])c([H])c1C2=O)c3c([H])c([H])c([Cl])c([Cl])c3[H]
33	[H]/C2=C(/Oc1c([H])c(O[H])c([H])c(O[H])c1C2=O)c3c([H])c([H])c(N(=O)=O)c([H])c3[H]
34	[H]/C2=C(/Oc1c(O[H])c(O[H])c([H])c([H])c1C2=O)C([H])([H])[H]
35	O=C2C(C(=O)c1c([H])c(OC([H])([H])[H])c([H])c([H])c1[H])=C(Oc3c([H])c(O[H])c([H])c([H])c23)c4c([H])c([H])c([H])c(OC([H])([H])[H])c4[H]
36	O=C2C(C(=O)c1c([H])c([H])c(N(=O)=O)c([H])c1[H])=C(Oc3c(O[H])c(O[H])c([H])c([H])c23)c4c([H])c([H])c(N(=O)=O)c([H])c4[H]
